# Brain Under Fatigue – Can Perceived Fatigability in Multiple Sclerosis Be Seen on the Level of Functional Brain Network Architecture?

**DOI:** 10.3389/fnhum.2022.852981

**Published:** 2022-05-10

**Authors:** Anna Maria Sobczak, Bartosz Bohaterewicz, Anna Ceglarek, Aleksandra Zyrkowska, Magdalena Fafrowicz, Agnieszka Slowik, Marcin Wnuk, Monika Marona, Klaudia Nowak, Kamila Zur-Wyrozumska, Tadeusz Marek

**Affiliations:** ^1^Department of Cognitive Neuroscience and Neuroergonomics, Institute of Applied Psychology, Jagiellonian University, Kraków, Poland; ^2^Department of Psychology of Individual Differences, Psychological Diagnosis, and Psychometrics, Institute of Psychology, University of Social Sciences and Humanities, Warsaw, Poland; ^3^Department of Neurology, Jagiellonian University Collegium Medicum, Kraków, Poland; ^4^Department of Neurology, University Hospital in Krakow, Kraków, Poland; ^5^Department of Medical Education, Jagiellonian University Medical College, Kraków, Poland; ^6^Department of Neurology, 5th Military Hospital, Kraków, Poland

**Keywords:** multiple sclerosis (MS), resting-state fMRI (rs-fMRI), fatigue, ALFF/fALFF, functional network (FN)

## Abstract

**Background:**

Fatigue is one of the most common symptoms of multiple sclerosis (MS), significantly affecting the functioning of the patients. However, the neural underpinnings of physical and mental fatigue in MS are still vague. The aim of our study was to investigate the functional architecture of resting-state networks associated with fatigue in patients with MS.

**Methods:**

The sum of 107 high-functioning patients underwent a resting-state scanning session and filled out the 9-item Fatigue Severity Scale (FSS). Based on the FSS score, we identified patients with different levels of fatigue using the cluster analysis. The low-fatigue group consisted of *n* = 53 subjects, while the high-fatigue group *n* = 48. The neuroimaging data were analyzed in terms of functional connectivity (FC) between various resting-state networks as well as amplitude of low-frequency fluctuation (ALFF) and fractional amplitude of low-frequency fluctuations (fALFF).

**Results:**

Two-sample *t*-test revealed between-group differences in FC of posterior salience network (SN). No differences occurred in default mode network (DMN) and sensorimotor network (SMN). Moreover, differences in fALFF were shown in the right middle frontal gyrus and right superior frontal gyrus, however, no ALFF differences took place.

**Conclusion:**

Current study revealed significant functional network (FN) architecture between-group differences associated with fatigue. Present results suggest the higher level of fatigue is related to deficits in awareness as well as higher interoceptive awareness and nociception.

## Introduction

Multiple sclerosis (MS) is an inflammatory, neurodegenerative disease characterized by demyelination and axonal damage in the central and peripheral nervous systems. There are four courses of the disease and the most common one is a relapsing-remitting form (RRMS), in which the relapses of symptoms are followed by periods of remission with a different duration and a break between them. The courses differ in the type of attack by the immune system, clinical picture as well as neurological symptoms (Filippi et al., 2018; Lassmann, 2018). The MR image, which is partly used for diagnosis, shows focal lesions that are triggered by chronic inflammatory processes and cause symptoms of the disease, including mobility problems, pain, and cognitive decline (Filippi et al., 2018). On a neuronal level, diseased and structurally injured brain causes functional disruption of various neural networks. Therefore, functional networks (FNs) can be characterized with different activity in early and late stages of the disease. Deficits and disruption in one network are reported to cause hyperconnectivity in another network, which compensate for the loss in the disrupted network. It is believed hyperconnectivity represents an observable brain response to neural network disruption (Hillary and Grafman, 2017). Functional compensatory mechanisms are especially reported to be very common at the early, mild stages of the MS, which can limit first clinical manifestations of the disease until a certain threshold of damage is exceeded (Pantano et al., 2002; Audoin et al., 2005; Mainero et al., 2006; Basile et al., 2014; Droby et al., 2016; Castellazzi et al., 2018; Koubiyr et al., 2021). One of the most common (occurring in up to 80% of patients) symptoms in the early stages of MS is fatigue, influencing normal functioning (Simionia et al., 2007; Forwell et al., 2008; Gupta et al., 2013; Loy et al., 2017). Rudroff et al. (2016) propose the model, where fatigue in MS is described as “the decrease in physical and/or mental performance that results from changes in central, psychological and/or peripheral factors.” Nevertheless, it differs significantly from tiredness seen in healthy individuals or other chronic disease patients and many MS patients describe fatigue as the most debilitating disease sign (Bakshi, 2003; Kos et al., 2007; Induruwa et al., 2012). The phenomenon of fatigue in MS can be understood on the level of state fatigue, which relates to psychological state in the specific moment of time or fatigue as a trait. Enoka et al. (2021) divide trait fatigue into perceived fatigability and objective fatigability. The former can be operationalized from capacity to perform past, present, and future activities, while the latter refers to limits of current actions, manifested in voluntary activations and contractile function.

The mechanisms of fatigue could be divided into primary ones such as involvement of immune system and altered patterns of brain activation through lesions or secondary mechanisms, which results from related conditions like sleep disorders or depression (Braley and Chervin, 2010). Nevertheless, the origin and mechanism of this symptom are still poorly understood. However, fatigue in MS is thought to be mostly linked to age, disability, and disease duration (Colosimo et al., 1995; Ghajarzadeh et al., 2013; Loy et al., 2017), with some suggestion that disability is a driving factor (Kroencke et al., 2000).

The researchers have been already looking for structural and functional correlates of fatigue in MS, however, metaanalyzes show that most of the studies focus on MS patients with moderate or severe disability status (Loy et al., 2017; Moss-Morris et al., 2021). So far, Wilting et al. (2015) using voxel-based morphometry revealed thalamic alterations associated with fatigue in the early stage of relapsing-remitting MS. Functional abnormalities of the brain could be distinguished by resting-state fMRI (rs-fMRI), which enables studying functional interactions in the state of rest, without any explicit task performance (Smith et al., 2013). These interactions and their alterations are determined in healthy individuals in various conditions and clinical approaches, including patients with MS. The previous research on MS indicated diminished synchronization in the cerebellum, thalamus, or insula with other brain regions (Zhou et al., 2013; Tona et al., 2014). Moreover, the alterations in functional connectivity (FC) in MS are correlated with the disability progression (Faivre et al., 2016; Tommasin et al., 2020). Zhou et al. (2016) also revealed disturbances in brain atrophy, a new approach to investigate brain temporal dynamics, in patients with MS compared to healthy subjects. Several studies associate resting-state FC with MS-related fatigue (e.g., Bisecco et al., 2017; Jaeger et al., 2018; Stefancin et al., 2019), demonstrating divergent results.

The disturbances of FC were so far revealed mainly in DMN (Bonavita et al., 2011, 2016; Zhou et al., 2014) and salience network (SN) (Cruz-Gómez et al., 2013; Manca et al., 2019). Moreover, the previous research revealed altered amplitude of low-frequency fluctuation (ALFF) in, i.e., motor, cerebellar, or frontal regions (Liu et al., 2011; Mansoory et al., 2018; Plata-Bello et al., 2018).

The aim of the current study is to determine the fatigue-related FC disturbances in default mode network (DMN), SN, and sensorimotor network (SMN) as well as whole brain ALFF and fractional amplitude of low-frequency fluctuations (fALFF) in the mild stage of RRMS. Based on the previous literature, we hypothesize hyperconnectivity in DMN, SN as well as SMN as a sign of compensation mechanisms in networks which are thought to be disrupted during the progression of MS. Moreover, we hypothesize altered ALFF, fALFF and graph measures in frontal, motor, and cerebellar regions.

## Materials and Methods

### Subjects

The recruitment strategy was based on a collaboration with the Multiple Sclerosis Treatment Center of Jagiellonian University Hospital. All patients meeting the criteria of relapsing-remitting type of the disease, the Expanded Disability Status Scale (EDSS) score no higher than 4, no flu-like symptoms, no contraindications for MRI scanning (pregnancy, use of electrostimulator or pacemaker, non-removable prosthesis in the body made from ferromagnetic material, claustrophobia) were invited to take part in the study. The invitation was given by their doctor during their routine, seasonal follow up visit. The Institute of Applied Psychology Ethics Committee of the Jagiellonian University approved the study, and an informed consent was obtained from all participants in accordance with the Declaration of Helsinki.

Due to the compromised quality of MRI scans, data from 6 participants needed to be removed from further analysis, the final research sample consisted of 101 participants (37 men) aged from 20 to 59 (*M* = 36.67; SD = 8.04). The mean EDSS score for all patients was *M* = 1.34; SD = 0.95.

### Basic Information and Short Medical Interview

Information about participants’ age and gender as well as their current EDSS score was provided by the recruiting physician. Short medical interview was conducted with each participant and information of the first occurrence of MS symptoms and the beginning of MS related treatment was gathered by the doctor.

Early, mild stage of the disease was defined due to the severity of the disease rather than due to the time from the beginning of the treatment or from the onset of first symptoms. As a results, doctors recruited patients whose EDSS score was no higher than 4 (*M* = 1.34; SD = 0.95). Patients up to that stage are self-sufficient and able to walk without rest or support for at least 500 m (Çinar and Yorgun, 2018). Above criteria are presumably more objective, since first onset of symptoms as recalled by patients can be biased and the beginning of treatment depends on the patient’s own subjective decision of when the symptoms were severe enough for them to seek medical advice. Moreover, after 10–20 years, many patients with relapsing-remitting MS develop secondary-progressive MS, where impairment accumulates over time (Moss-Morris et al., 2021). Above fact may contribute to the statement that current study investigates MS patients in early stages.

### Fatigue Severity Scale Assessment

All participants filled in the Fatigue Severity Scale (FSS) (Krupp et al., 1989). The method is a one-dimensional questionnaire consisting of nine statements about the feeling of fatigue experienced during last week. Seven-point Likert scale is used to rank the level of agreement with every statement from 1 – strongly disagree to 7 – strongly agree. Overall score is a sum of all questions divided by nine. Score of 4 and greater is considered to indicate a substantial level of fatigue (Andreasen et al., 2011; Learmonth et al., 2013). Fatigue Severity Scale is a method widely used in studies on MS and is characterized with good psychometric properties with Cronbach’s alpha of 0.93 (Amtmann et al., 2012). According to Enoka et al. (2021) it allows to quantify perceived fatigability.

In the current study participants filled out Polish version of the questionnaire used previously in another study conducted on Polish population (Goła̧b-Janowska et al., 2016). Scale was translated from English by a team consisting of a neurologist and a translator and then back-translated by an independent bilingual person. Any inaccuracies were discussed and corrected.

### Expanded Disability Status Scale

Prior to the study all participants were assessed on the EDSS which is a tool widely used to measure disability level in patients with MS (Çinar and Yorgun, 2018). It is based on structured interview and clinical examination. The final score of the scale ranges from 0 – normal neurological examination to 10 – death.

### MRI Acquisition

MRI data was acquired using 3T Siemens Skyra MR System (Siemens Medical Solutions, Erlangen, Germany). Structural images were obtained using sagittal 3D T1-weighted MPRAGE sequence. Total of 13 min and 20 s rs-fMRI EPI images were acquired using gradient-echo single-shot echo planar imaging sequence with the following parameters: TR = 800 ms; TE = 27 ms; slice thickness = 0.8 mm, voxel size = 3 mm^3^, with no gap using 60-channel coil. Total of 52 interleaved transverse slices and 1000 volumes were acquired. During the acquisition, participants were instructed to keep their eyes open and not to think about anything in particular. Simultaneous-multi-slice (SMS) acquisition was acquired in order to enhance the sensitivity of hemodynamic response by acquiring two or more slices simultaneously and as a consequence, decreasing TR to 0.8 s.

### Imaging Data Preprocessing

The rs-fMRI data processing was performed using Data Processing & Analysis for Brain Imaging (DPABI) V6.0 (Yan et al., 2016) as well as SPM 12 (Wellcome Trust Centre for Neuroimaging, UCL, London, United Kingdom) both working under MATLAB version R2018a (The MathWorks, Inc., Natick, MA, United States). Firstly, 10 time points were discarded due to signal equilibration and then slice timing was conducted. Next, realignment with assessment of the voxel specific head motion was conducted. Six participants displayed movements above 3 mm or 3° in one or more of the orthogonal directions and therefore had to be disqualified from further analysis. Then, using standard EPI template functional images were linearly normalized in DARTEL to Montreal Neurological Institute (MNI) space and spatially resampled to 2 × 2 × 2 mm voxel size. The 24 motion parameters derived from the realignment step, white matter as well as cerebrospinal fluid signals and five principal components were removed using principal components analysis integrated in a component based noise correction method (Behzadi et al., 2007). The global signal was included due to its potential to provide additional valuable information (Liu et al., 2017). The signal was then band-pass filtered (0.01–0.08 Hz) to reduce high-frequency noise and low-frequency drift, such as the respiratory and cardiac rhythms. The data was not smoothed.

### *K*-Means Clustering

*K*-means is considered to be the mostly used clustering technique for vector data (Thirion et al., 2014). It results in the partition of *n* observations into *k* clusters, where each observation belongs to the cluster with the nearest mean, described as cluster centers. In current study, supervised *k*-means algorithm was used. Participants were divided into 2 clusters according to overall FSS score. According to the literature, the score of fatigue which is 4 or higher is considered to indicate substantial level of fatigue (Andreasen et al., 2011; Learmonth et al., 2013). As a result of *k*-means clustering, a low-fatigue group (*M* = 2.41; SD = 0.74) consisted 53 subjects while the high-fatigue group (*M* = 4.64; SD = 0.77) had 48 subjects in it.

### Analyzes Rationale

In order to investigate functional architecture of neural networks, three types of fMRI analyzes were conducted. Firstly, FC within DMN, SN, and SMN was calculated. Secondly, whole-brain analyzes were explored. Conventional (ALFF) and fALFF were calculated with the use of typical, universal frequency band of 0.01–0.08 Hz. Moreover, topological properties of FN were investigated on a global and local level with the use of graph metrics.

### Functional Connectivity Analysis

First level FC analysis was calculated within five sets of regions using an ROI-to-ROI approach. Five regions of interests (ROIs), such as DMN, SN, and SMN were chosen based on the regions defined by Stanford University. All ROIs can be downloaded in author’s website.^1^ ROIs include: dorsal DMN, ventral DMN, posterior SN, anterior SN and SMN. Raw time courses were extracted from each subject using “ROI Signal Extractor” module in DPABI V6.0 (Yan et al., 2016) working under MATLAB version R2018a (The MathWorks, Inc., Natick, MA, United States) and SPM 12 (Wellcome Trust Centre for Neuroimaging, UCL, London, United Kingdom). The two-sample *t*-tests comparing FC values in both groups were performed for every ROI, the significance level was set to *p* < 0.05, with FDR correction (Benjamini–Hochberg procedure) for multiple comparisons.

### Amplitude of Low-Frequency Fluctuation and Fractional Amplitude of Low-Frequency Fluctuations Analysis

Amplitude of low-frequency fluctuation allows to estimate the neural component from the BOLD signal, showing the content of the power is in the low-frequency range. ALFF is a whole-brain analysis, which enables focusing on each voxel of the brain, making it a complementary method to FC (Wang et al., 2016).

Fractional amplitude of low-frequency fluctuations, on the other hand, measures the power within a specific frequency range divided by the total power in the entire detectable frequency range which is 0–0.25 Hz (Zou et al., 2008). Noteworthy, it is considered as more sensitive to neural origins of low-frequency fluctuations (Bijsterbosch and Beckmann, 2017), making the analysis of low frequency fluctuations more comprehensive.

Amplitude of low-frequency fluctuation as well as fALFF were calculated using DPABI v 6.0 (Yan et al., 2013). The time series for each voxel was transformed to the frequency domain with the use of a fast Fourier transform. The square root of the power spectrum was calculated, then averaged across 0.01–0.08 Hz, and standardized to z-score by dividing the subject-level maps by the standard deviation. For standardization purposes and to reduce the influence of individual variation in ALFF values, the ALFF of each voxel was then divided by the global mean of ALFF values for each subject within the default brain mask from the DPABI, removing background and other signals which were not from brain tissue. As a result, a standardized whole-brain ALFF map was created. Both ALFF and fALFF were calculated in the typical frequency band of 0.01–0.08 Hz. ALFF and fALFF statistical analyses were conducted at the voxel-level of *p* = 0.001.

### Graphs Metrics

GraphVar 2.02b (Kruschwitz et al., 2015) and MATLAB version R2018a (The MathWorks, Inc., Natick, MA, United States) were used in order to examine the topological properties of functional brain network at global and local levels. Global measures were used to describe macroscale organization and integration of all nodes in the brain network and included: mean clustering coefficient and assortativity. Local properties were calculated for each individual node (region) separately and reflecting the nodal centrality in the network. In this study, common local properties such as clustering coefficient and eigenvector centrality were calculated (the measures are discussed in detail in https://sites.google.com/site/bctnet/measures/list). Data used for graph measures were not smoothed during preprocessing steps. For each subject, 116 ROIs were defined according to the AAL atlas (Tzourio-Mazoyer et al., 2002). In order to obtain a 116 × 116 undirected binary correlation matrix, mean time course for each region was extracted and then the Pearson coefficients between each pair of ROIs were calculated. In order to exclude the spurious links in interregional connectivity matrices (Power et al., 2011), we adopted a thresholding procedure based on the strongest connections, removing the weaker ones (van den Heuvel et al., 2017). Above procedure enabled to compare network topology between as well as within participants (Gamboa et al., 2014). Network edges were defined using a sparsity thresholding procedure ranging from 0.1 to 0.5 in steps of 0.05, universal for all of the subjects. Both age and EDSS scores were used as a nuisance covariate.

## Results

Cluster 1 consisted of 53 patients (20 men) aged from 20 to 56 (*M* = 35.06; SD = 7.87). Mean FSS score (*M* = 2.41; SD = 0.74), EDSS score (*M* = 1.15; SD = 0.79), years since onset of first MS symptoms (*M* = 8.06; SD = 4.73) as well as years since the start of MS related treatment (*M* = 5.43; SD = 3.23) were calculated. Cluster 2 consisted of 48 patients (17 men) aged from 25 to 59 (*M* = 38.46; SD = 7.91). Mean FSS score (*M* = 4.64; SD = 0.77), EDSS score (*M* = 1.54; SD = 1.06), years since onset of first MS symptoms (*M* = 9.81; SD = 4.62), and years since the start of MS related treatment were calculated (*M* = 6.98; SD = 3.5). Clusters differed significantly in age [*t*_(2,99)_ = 2.16; *p* = 0.033] and the older patients were placed in the Cluster 2. Moreover, patients from Cluster 2 also obtained significantly higher scores in FSS [*t*_(2,99)_ = −14.79; *p* < 0.001], EDSS [*t*_(2,99)_ = 2.083; *p* = 0.04], and were treated for MS longer than participants from Cluster 1 [*t*_(2,99)_ = 2.308; *p* = 0.023]. Clusters showed no significant difference in gender χ^2^ = (1, *n* = 101) = 0.058; *p* = 0.809 and years from the first onset of MS symptoms *t*_(2,99)_ = 1.882; *p* = 0.063. All demographic values are showed in Table 1.

**TABLE 1:** Demographic and medical data of patients in clusters.

	Cluster 1	Cluster 2	Test result	*p*
Gender	M:20 F:33	M:17 F:31	χ^2^ = (1, *n* = 101) = 0.058	0.809
FSS	*M* = 2.41 SD = 0.74	*M* = 4.64 SD = 0.77	*t*_(2,99)_ = 14.79	< 0.001
Age	*M* = 35.06 SD = 7.87	*M* = 38.46 SD = 7.91	*t*_(2,99)_ = 2.16	0.033
EDSS	*M* = 1.15 SD = 0.79	*M* = 1.54 SD = 1.06	*t*_(2,99)_ = 2.083	0.04
Years since the onset of first symptoms	*M* = 8.06 SD = 4.73	*M* = 9.81 SD = 4.62	*t*_(2,99)_ = 1.882	0.063
Years since the start of treatment	*M* = 5.43 SD = 3.23	*M* = 6.98 SD = 3.5	*t*_(2,99)_ = 2.308	0.023

**FIGURE 1:**
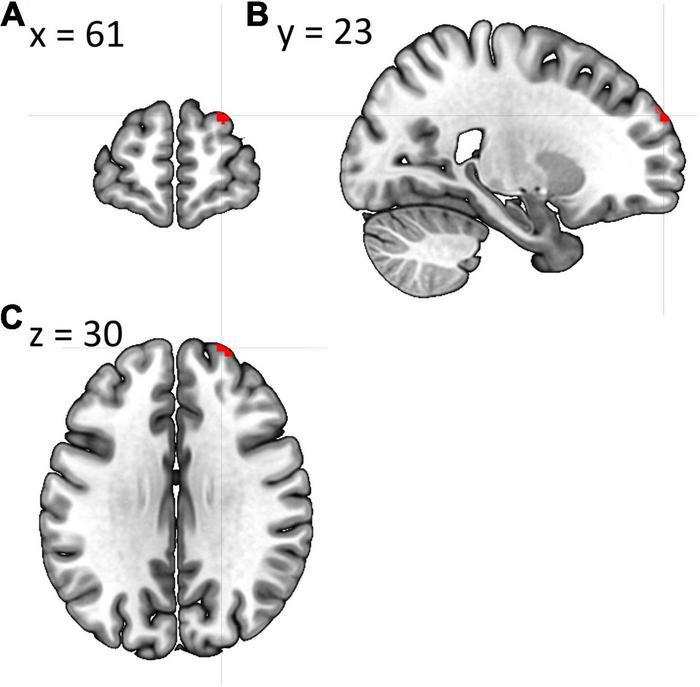
Cluster showing whole-brain fALFF analysis differences between both groups in **(A)** coronal plane, **(B)** sagittal plane, and **(C)** axial plane. The template “Ch2bet” was obtained from MRIcroGL.

**FIGURE 2:**
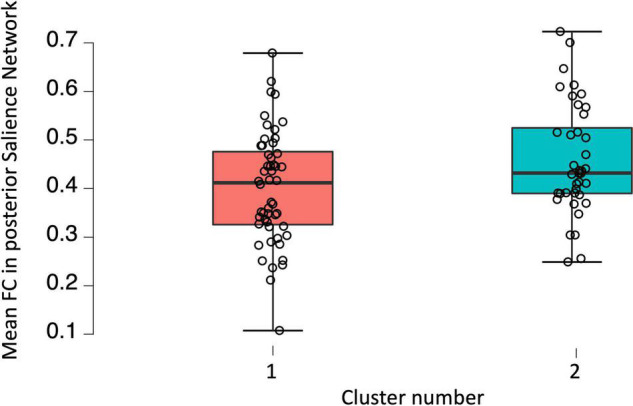
Boxplot showing raw functional connectivity values in posterior salience network for groups with low and high fatigue.

A one-way ANCOVA was conducted to investigate potential between-group differences in FC of DMN, SN, and SMN associated with the level of fatigue. The analysis revealed statistically significant difference between patients in Cluster 1 and Cluster 2 in posterior SN, while controlling for age, disability level, and duration of pharmacological treatment [*F*_(2,90)_ = 6.093; *p* = 0.016]. *Post hoc* comparisons using Bonferroni test indicated that the mean FC values for the Cluster 1 (*M* = 0.401; SD = 0.115) was significantly different from mean FC values for Cluster 2 (*M* = 0.457; SD = 0.113). These results suggests that the FC between brain areas among posterior SN was higher in Cluster 2 in which the fatigue level was increased as compared to Cluster 1 and that this result is independent of age, disability level, and duration of pharmacological treatment. A one-way ANCOVA showed no significant differences between Cluster 1 and Cluster 2 in FC among anterior SN [*F*_(2,91)_ = 1.858; *p* = 0.176], dorsal [*F*_(2,91)_ = 1.353; *p* = 0.248], and ventral [*F*_(2,91)_ = 0.906; *p* = 0.344] parts of the DMN as well as SMN [*F*_(2,91)_ 0.590; *p* = 0.444]. Furthermore, two sample *t*-test showed statistically significant between-group differences in fALFF [*t*_(2,99)_ = 3.17; *p* = 0.027]. The threshold was set at the *p*-value 0.001 with cluster size of 28 voxels. Patients with higher fatigue level had lower fALFF in right middle frontal gyrus as well as right superior frontal gyrus. No differences were found in ALFF as well as graph measures (*p* > 0.05). All of the fMRI results are FDR corrected with *p* < 0.05. Visualization of above results can be found in Figures 1, 2.

## Discussion

The aim of the current study was to investigate the association of FN architecture in DMN, SN, and SMN as well as whole-brain ALFF and fALFF with the level of fatigue in the mild stage of RRMS.

The investigation revealed higher average FC in posterior SN in Cluster 2 in comparison with Cluster 1. The SN play an enormous role in attentional processes such as its distribution and capacity (Manca et al., 2019), enabling productive information processing as well as faster responses to stimuli. At the same time, people with chronic fatigue syndrome are coherently described as the ones with significant deficits in attention (Ray et al., 1993; Holgate et al., 2011). Accordingly, subjects from Cluster 2, who declare to be almost twice as much tired as participants from Cluster 1, manifest decreased attention, compared to the group with the lower scores of fatigue. Therefore, higher FC in posterior SN in RRMS patients might be a compensatory mechanism which enables the patients to cope with deficits of attention. Many studies point to the compensation mechanisms in MS patients, especially in the early stages (Pantano et al., 2002; Audoin et al., 2005; Mainero et al., 2006; Basile et al., 2014; Droby et al., 2016; Castellazzi et al., 2018; Koubiyr et al., 2021). Hyperconnectivity is thought to represent an observable brain response to neural network disruption caused by diseased and structurally injured brain (Hillary and Grafman, 2017). However, only short-term hyperconnectivity is considered to be beneficial, while long-term hyperconnectivity can have bad consequences for human brain. Increased brain metabolism, caused by a local hyperconnectivity, is a predictor of higher amyloid beta (Aβ) deposition, which is a well-known signature of Alzheimer’s disease. In conclusion, chronic hyperconnectivity, performing a function of compensatory mechanisms, can lead to a serious progressive neurodegeneration (Vlassenko et al., 2010; Myers et al., 2014; Hillary and Grafman, 2017). Noteworthy, the main region of the posterior SN is posterior insula, which is widely known to be involved in interoceptive awareness, understood as a capability to knowingly perceive inner bodily signals (Kuehn et al., 2016). According to Craig et al. (2000), changes in the activity of posterior insula correlate with the graded intensity of interoceptive stimuli, while stimulation of the same region of interest results in interoceptive and somatic sensations (Penfield and Faulk, 1955). Moreover, posterior insula is thought to play a crucial role in nociception (Frot et al., 2014; Segerdahl et al., 2015). Higher nociception in MS patients stays in line with the literature, as chronic neuropathic pain is frequent and very characteristic symptom of this neurodegenerative disease (Yilmazer et al., 2020). Current study is also congruent with the research of Chen et al. (2020), who observed increased FC in the parts of insula which were described as an interoceptive hub. What is more, the study concludes that hyperconnectivity of interoceptive network is associated with experiencing of the cognitive fatigue.

Another result from the current study revealed smaller fALFF in RRMS patients with higher fatigue score in the cluster located in right middle frontal gyrus and right superior frontal gyrus. In the study of Sepulcre et al. (2009) lesion load in the exact same regions was positively correlated with the fatigue score. Authors concluded that lesions in aforementioned regions are associated with the disruption of FNs responsible for cognitive and attention processes. Other studies also point out the relation of middle frontal gyrus and fatigue in MS (Filippi et al., 2002; Calabrese et al., 2010). Noteworthy, both right middle frontal gyrus as well as the right superior frontal gyrus are thought to be located at the borderline between DMN and cognitive executive network (Young et al., 2017), while the SN is reported to modulate the switch between aforementioned two networks (Menon, 2015). Above results are very promising, considering hyperconnectivity in SN in MS patients with higher fatigue.

Noteworthy, brain regions with significantly altered FC as well as fALFF are anatomically or functionally connected to striatal-thalamic-frontal network, commonly known as the fatigue network (Genova et al., 2013). Genova et al. (2013) with the use of cognitively fatiguing task and FSS revealed that striatum as well as thalamus, frontal regions and its interconnected regions play significant role in perceived fatigability in MS patients. Interestingly, the SN, which had higher FC in patients with higher fatigue, consists regions which shows intrinsic connection to striatum and thalamus (Menon, 2015). The same group of patients manifested smaller fALFF in middle and superior frontal gyrus, which overlap with the striatal-thalamic-frontal network. Therefore, our results are partly congruent with the results of Genova et al. (2013) pointing on neural correlates of perceived fatigability in MS.

The current study revealed that MS patients with higher fatigue level were characterized with significantly higher disability status, age as well as longer pharmacological treatment. Above results are congruent with various previous studies (Colosimo et al., 1995; Kroencke et al., 2000; Ghajarzadeh et al., 2013) as well as the meta-analysis of Loy et al. (2017), who reports that MS patients with higher EDSS and longer disease duration suffer from fatigue more than patients with lower EDSS. Aforementioned questionnaire results, which stays in line with other studies in the field, strengthen the fMRI results on the neural basis of fatigue in mild stage MS.

### Limitations

The study has potential limitations. For example, using supervised *k*-means algorithm for dividing patients into clusters, as *k*-means was traditionally used for the unsupervised clustering. However, using unsupervised *k*-means clustering is not guaranteed to group the same types of objects together. Therefore, some supervision is needed to select objects which have the same label into one cluster. It has also been shown that the supervised *k*-means algorithm can be efficiently and effectively applied to various data sets (Al-Harbi and Rayward-Smith, 2006), hence we believe that using the supervised *k*-means algorithm was appropriate for current study. Secondly, the EDSS has some limitations. It provides ordinal scores and therefore contains less information than a scale using continuous measures, such as for example Multiple Sclerosis Functional Composite (MSFC) (Rudick et al., 2002). It was also reported that MSFC correlates better with brain measures and quality of life in MS patients (Kalkers et al., 2001; Rudick et al., 2002). However, Meyer-Moock et al. (2014) compared in their comprehensive study psychometric properties of both forementioned scales and concluded that both instruments display satisfying parameters. They pointed out that EDSS have an advantage in being internationally accepted and widely used. In addition, future studies should consider recruiting patients with the late stage of MS, in order to compare both stages with each other and broaden the picture of neuronal bases of fatigue in MS. Furthermore, we examined patients who could be treated with the medications which cause symptoms similar to the fatigue. Future studies should also measure pain, anxiety, and depressive symptoms as they are significant part of MS.

In conclusion, current study revealed significant FN architecture between-group differences associated with the fatigue. Present results suggest that the higher level of fatigue is related to deficits in attention as well as higher interoceptive awareness and nociception.

## Data Availability Statement

The raw data supporting the conclusions of this article will be made available by the authors, without undue reservation.

## Ethics Statement

The studies involving human participants were reviewed and approved by the Institute of Applied Psychology Ethics Committee of the Jagiellonian University. The patients/participants provided their written informed consent to participate in this study.

## Author Contributions

MF, TM, MW, AMS, BB, AC, and AZ: conception and design of the work. AMS, BB, AC, and AZ: data acquisition. AMS, BB, and AC: rs-fMRI data analysis. AMS, BB, MF, and TM: interpretation of the results. AMS, BB, AC, AZ, MW, AS, MM, KN, and KZ-W: drafting the work. AMS, BB, AC, AZ, MF, TM, MW, AS, MM, KN, and KZ-W: revising. MW, AS, MM, KN, and KZ-W: patients recruitment and diagnosis, revising the manuscript critically and final approval of the version to be published. All authors contributed to the article and approved the submitted version.

## Conflict of Interest

The authors declare that the research was conducted in the absence of any commercial or financial relationships that could be construed as a potential conflict of interest.

## Publisher’s Note

All claims expressed in this article are solely those of the authors and do not necessarily represent those of their affiliated organizations, or those of the publisher, the editors and the reviewers. Any product that may be evaluated in this article, or claim that may be made by its manufacturer, is not guaranteed or endorsed by the publisher.

## Footnotes

1
https://findlab.stanford.edu/research
